# SBRT for Colorectal Cancer Liver Metastasis Is a Safe and Effective Treatment Option—A Single Institution Retrospective Analysis

**DOI:** 10.3390/cancers18182917

**Published:** 2026-09-09

**Authors:** Nitsan Peled Oved, Marc Wygoda, Adi Levy, Mor Oved, Philip Blumenfeld, Ayala Hubert, Aron Popovtzer, Tamar Peretz, Aviad Zick, Tal Falick Michaeli

**Affiliations:** 1 Department of Medical and Radiation Oncology, Sharett Institute of Oncology, Hadassah Medical Center, Jerusalem 91120, Israel; nitsan.peled@gmail.com (N.P.O.); mwygoda@hadassah.org.il (M.W.); adi.health@gmail.com (A.L.); mor.oved@mail.huji.ac.il (M.O.); philipb@hadassah.org.il (P.B.); ayalah@hadassah.org.il (A.H.); aron@hadassah.org.il (A.P.); tamary@hadassah.org.il (T.P.); 2 Department of Military Medicine and “Tzamert”, Faculty of Medicine, Hebrew University of Jerusalem, Jerusalem 91120, Israel; 3 Department of Developmental Biology and Cancer Research, Institute for Medical Research Israel-Canada, Hebrew University Medical School, Jerusalem 91120, Israel

**Keywords:** colon cancer, radiotherapy, SBRT, metastasis resection, hepatic metastases from colorectal cancer

## Abstract

Background: Colorectal cancer is the third most commonly diagnosed cancer in the world. Liver metastases are frequent, adversely affecting patient prognosis. We compared progression-free survival and overall survival in colorectal cancer patients with liver metastasis treated with surgical resection or stereotactic body radiotherapy. No previous study had shown superiority of one treatment option over the other. Methods: We conducted a retrospective cohort study of 40 colorectal cancer patients with liver metastasis treated with surgical resection or stereotactic body radiotherapy at the Hebrew University-Hadassah Medical Center. Patient demographics, tumor characteristics, treatment details, and survival outcomes were analyzed. Kaplan–Meier curves and log-rank tests were used for survival analysis. Results: The median overall survival for the entire cohort was 44 months (95% CI 29–59 months). In patients treated with surgical resection, the median overall survival was 51 months (95% CI: 36–67 months), while in patients treated with stereotactic body radiotherapy the median overall survival was 32 months (95% CI: 21–43 months), a non-statistically significant result (*p* = 0.306). The number of lobes involved did not significantly impact overall survival (*p* = 0.214). Additionally, it did not significantly affect progression-free survival (*p* = 0.41). Liver metastases located in the left lobe led to considerably worse progression-free survival compared to other regions involved, specifically in the patients who underwent surgery. Conclusions: Our findings underscore the importance of personalized treatment strategies tailored to the distinct characteristics of colorectal cancer patients with liver metastasis. Currently, surgical resection remains the standard of care. Further research is warranted to address the possibility of extending SBRT to first-line treatment in selected unresectable patients.

## Simple Summary

Colorectal cancer often spreads to the liver, and the only potentially curative treatments are surgery and a precise form of radiotherapy called stereotactic body radiotherapy (SBRT). It was not clear whether one treatment offers a survival advantage over the other. We reviewed the records of 40 patients treated with either surgery or SBRT for liver metastases from colorectal cancer at a single medical center. Overall survival was numerically longer after surgery than after SBRT, but the difference was not statistically significant. Patients whose tumors involved more than one part of the liver had a higher risk of disease progression, and tumors located in the left part of the liver were linked to more progression regardless of treatment. These findings support personalizing treatment choice for each patient and suggest SBRT may be a reasonable option for patients who cannot undergo surgery.

## 1. Introduction

Colorectal cancer is the third most diagnosed cancer among men and the second among women worldwide, accounting for an estimated 1.4 million new cases and 693,900 deaths annually, and it ranks third among cancer-related causes of death [1]. In population-based studies, 25–30% of colorectal cancer patients have metastatic spread to the liver (CRLM) [2]. Management of CRLM is challenging and requires multidisciplinary discussion. Physicians assess the patient’s likelihood of cure and select a treatment approach based on the number, size, and location of the identified lesions.

Treatment options include surgical and non-surgical local therapies and tailored systemic therapy [3]. At present, surgical intervention and liver-directed therapies such as ablation and radiotherapy constitute the cornerstone of curative strategies for CRLM [4]. Resection of CRLM is advocated for all patients deemed resectable, with 5-year survival rates exceeding 40% [4]. However, only 15% of patients are resectable at presentation [5]. Surgical outcomes are most favorable in patients with solitary liver metastases, but overall survival (OS) is still longer in patients with multiple metastases who undergo resection as opposed to those left unresected [4,6,7]. Patients eligible for resection must meet at least one of two main criteria: first, sparing of at least two adjacent liver segments with independent inflow, outflow, and biliary drainage. Additionally, the residual liver volume must not fall below 20% and 30% of the total liver volume in non-cirrhotic and cirrhotic patients, respectively [2,8,9,10]. There is a wide range of contraindications for surgery, including the patient’s performance status, metastatic distribution or location, and limited liver reserve [5]. Surgical complications can vary depending on the extent of resection, patient factors, and perioperative care. These complications are divided into acute, general surgical complications or late effects including tumor recurrence [11,12].

Although previously used only for palliative purposes, radiation therapy (RT) has been transformed over the past two decades by sophisticated treatment planning software and advances in image-guided techniques. Stereotactic body radiation therapy (SBRT) now enables the delivery of higher radiation doses in fewer fractions to discrete liver lesions while sparing healthy hepatic tissue [3]. The delivery of ablative doses has led to significantly improved local control with RT [13,14]. An early multi-institutional phase 1/2 trial of SBRT for CRLM demonstrated a 2-year local control (LC) rate of 92% and a median OS of 20.5 months. In lesions with a maximal diameter of ≤3 cm (n = 38), the 2-year LC was 100% [15]. Several factors need to be considered to ensure safety, including presence of sufficient reserve of non-irradiated liver, good liver function, and tumor location being distant from luminal gastrointestinal tissues, so that the doses of SBRT can be delivered while avoiding potential toxicity [16]. Better outcomes are noted in patients with smaller lesion size, limited extrahepatic disease, a limited number of hepatic lesions, and dose escalation [3,16]. SBRT toxicity varies and includes liver-related toxicity, of which Radiation-Induced Liver Disease (RILD) is the most life-threatening; however, the risk is relatively low, especially in patients with good liver function. Overall, SBRT has been shown to be a tolerable and safe treatment with reliable oncologic outcomes in patients with CRLM [17,18,19].

Resection of CRLM is the standard of care. To our knowledge, no previous study has directly compared SBRT with resection. Here we retrospectively compare the oncologic outcomes of SBRT with resection of CRLM. Additionally, we report the impact of the anatomical distribution of liver metastases on progression-free survival (PFS) after resection or radiation therapy.

## 2. Materials and Methods

### 2.1. Study Design and Patient Eligibility

In this retrospective single-center study, we assessed treatment effectiveness for CRLM according to metastasis location and treatment modality in patients with stage IV colorectal cancer treated at Hadassah Medical Center between 2014 and 2024. Inclusion criteria included: (1) diagnosis of stage IV adenocarcinoma of the colon,(2) metastatic spread to the liver, and (3) treatment with either surgery or SBRT. No patients with rectal cancer as the primary tumor site met the inclusion criteria.

**Figure 1:**
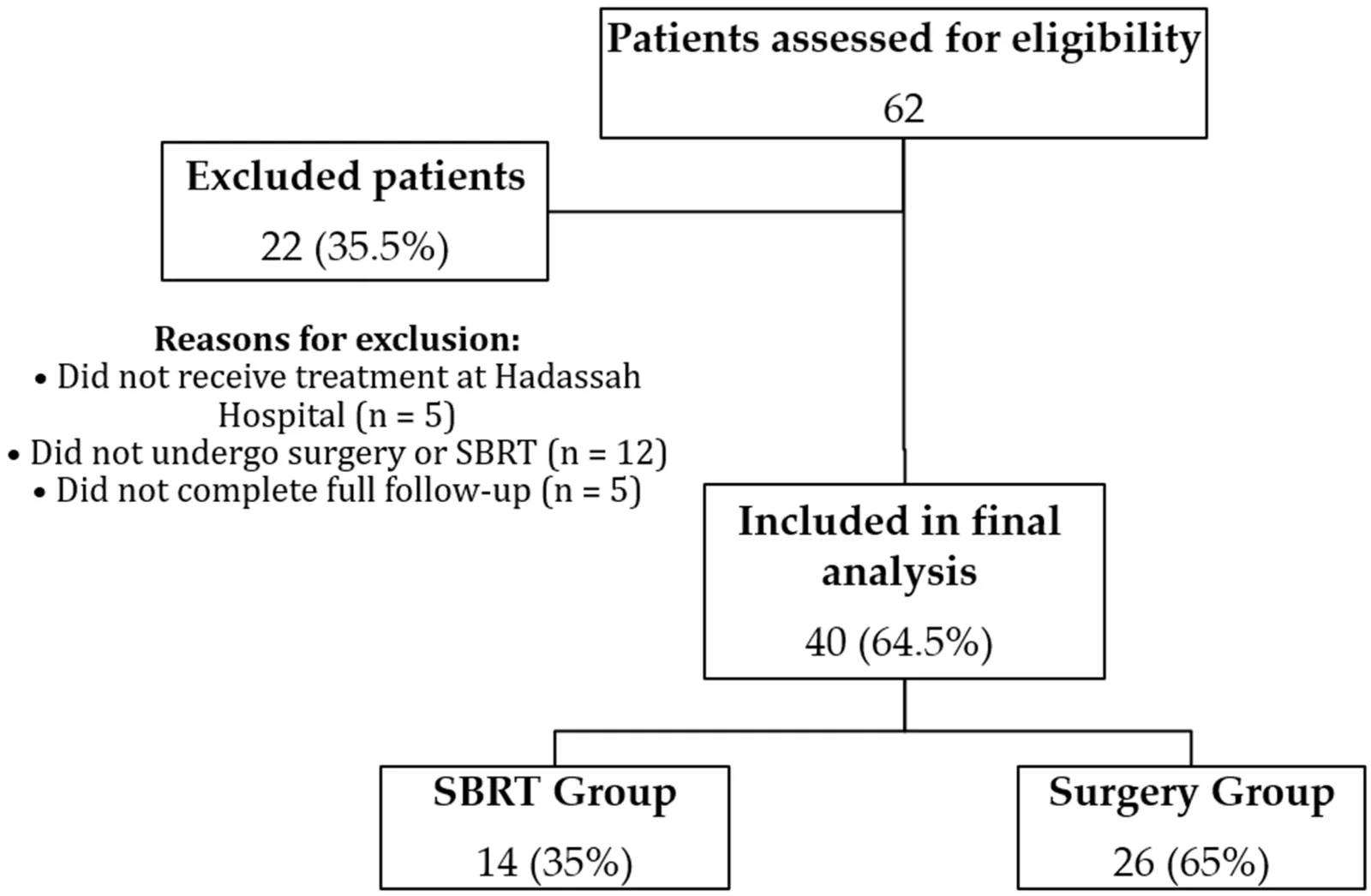
Consort diagram.

Out of 62 patients initially meeting our inclusion criteria, 22 cases were excluded as shown in Figure 1, based on the following criteria: (1) 5 patients who did not receive treatment at Hadassah Hospital, resulting in missing information; (2) 12 patients who either did not undergo surgical resection of the metastases or were treated with RT with palliative intent; (3) 5 patients who did not complete full follow-up, making it impossible to determine treatment outcomes.

For data collection, we used the “MAHAR” database, the electronic medical record system used at Hadassah Ein Kerem Hospital, which contains the patients’ medical records. Data from MAHAR were manually entered into a REDCap platform used to manage records of patients enrolled in the study. The REDCap database contains the patient’s ID number, demographic information, details about the nature of the patient’s tumor, and information about the treatments received. The research also relied on the database of the “Varian v18.1” software, which is used at Hadassah Medical Center for planning and managing radiotherapy treatment for patients at the “Sharett” Oncology Institute.

The collected data included demographic details of the patients, pathological subtype of the primary tumor, location of liver metastases, date of the last follow-up visit or date of death, disease recurrence and its date, date of metastasis appearance, and the treatment chosen for metastasis removal. Among patients who underwent metastasis resection, the surgery date and adjuvant systemic treatments received were examined. Among patients who underwent targeted radiation therapy, the duration of treatment, number of fractions, cumulative dose intensity, and adjuvant systemic treatments received were investigated.

The study was conducted in accordance with the Declaration of Helsinki and was approved by the Hadassah Medical Center IRB (protocol code 0346-12, 14 November 2021).

### 2.2. Study Groups

Patients were divided into groups based on the treatment modality selected by the physician after multidisciplinary discussion. These groups included surgical resection and SBRT. Liver resection is the standard of care; as such, radiation treatments were delivered only to patients with oligometastases in the liver who were not eligible for surgery. For classification of metastasis location in the liver, we used the Couinaud classification of liver anatomy. This classification divides the liver into eight functionally independent segments. Each segment has its own vascular inflow, outflow, and biliary drainage. The left lobe comprises segments II–IV, while the right lobe comprises segments V–VIII. Segment I consists of the caudate and quadrate lobes [6].

### 2.3. Radiotherapy Treatment Characteristics

SBRT is an advanced radiotherapy technique enabling the delivery of higher radiation doses to localized lesions. Prescription dose was defined as follows; The 100% isodose surface encompassed at least 95% of the planning target volume (PTV). Critical organ-at-risk (OAR) dose constraints were met in all standard plans. In scenarios where full target coverage compromised OAR limits, a clinical trade-off favoring organ sparing over PTV coverage was applied at the radiation oncologist’s discretion. The biologically effective dose (BED) was calculated assuming a standard α/β ratio of 10 Gy.

Respiratory motion was accounted for using an internal target volume (ITV) strategy. At simulation, a 10-phase 4D-CT scan was acquired to capture tumor kinematics throughout the entire respiratory cycle. An ITV encompassing the full range of tumor excursion was subsequently delineated. Although this approach expands the overall treatment volume and increases non-target tissue exposure, it guarantees continuous target coverage irrespective of respiratory phase.

### 2.4. Disease Management and Follow-Up

CT and MRI were used to assess the location of the metastases in the liver and to evaluate the resectability of the metastases, according to the protocol [3]. Resection was performed on all resectable patients. Progression-free survival (PFS) was calculated from the date of the surgery or first SBRT session to the time of detected progression. Patients were followed every 3–6 months, with abdominal CT or MRI and clinical assessments.

### 2.5. Outcomes

Primary outcomes were defined as local progression of the metastases in the liver (progression-free survival—PFS) and death from any cause (overall survival—OS). Regional PFS was defined as the time interval between the date of surgery or the first course of SBRT and the appearance of new metastases or disease progression shown on liver imaging. OS was calculated as the time from surgery or the first SBRT treatment to death or last follow-up.

### 2.6. Statistical Methods

The quantitative variables collected in the study are presented as mean and standard deviation. Categorical variables are presented as the proportion of each category out of the total number of patients in the study. The Kaplan–Meier model was to analyze the effect of categorical variables on PFS or OS, with the log-rank test for comparing survival curves. The Cox regression model was applied to test the effect of quantitative variables on PFS and OS. This model was also used for multivariate survival analysis, yielding hazard ratios (HR) with 95% confidence intervals (CI). All statistical tests were two-tailed, and a *p*-value of 5% or less was considered statistically significant.

## 3. Results

### 3.1. Patients’ Characteristics

We gathered clinical data on 40 patients who underwent treatment for CRLM, with surgical intervention accounting for 65% of cases while 35% of patients underwent radiation therapy. The patients’ demographic and tumor characteristics are summarized in Table 1, and treatment details are summarized in Table 2.

### 3.2. Progression-Free Survival Is Similar in Colorectal Cancer Patients with Liver Metastasis Treated with Either Surgery or Radiotherapy

The median duration of PFS across all study groups was 10 months (95% CI 1.10–19.72 months). In addition, regional control, defined as the absence of progression within the liver, was achieved in 16 patients (40%) during the follow-up period.

**Figure 2:**
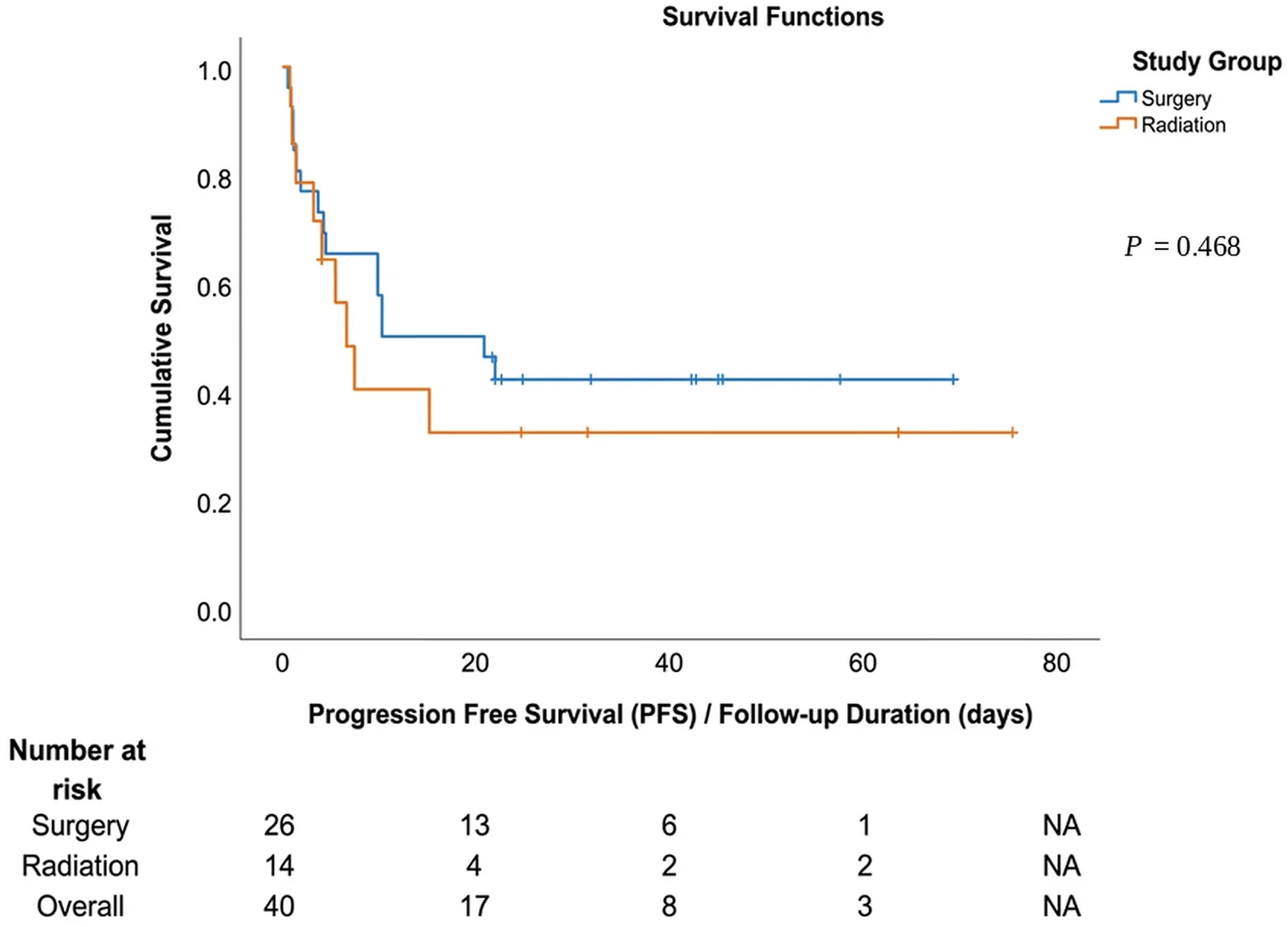
Progression-free survival according to treatment (study groups). Progression-free survival is similar in colorectal cancer patients with liver metastasis treated with either surgery or radiotherapy. cum = cumulative. *p* value = 0.468.

In subgroup analysis, distinct PFS profiles emerged within each treatment cohort, as shown in Figure 2. Among patients undergoing surgical resection, the median PFS was 10 months (95% CI: 0–25.21 months), whereas those treated with SBRT exhibited a median PFS of 8 months (95% CI: 3.37–10.09 months). Comparative analysis utilizing log-rank tests showed no statistically significant association between treatment modality and PFS across both study groups (*p* = 0.468) (Figure 2).

### 3.3. Overall Survival Is Numerically Increased in Colorectal Cancer Patients with Liver Metastasis Treated with Surgery Compared to SBRT

**Figure 3:**
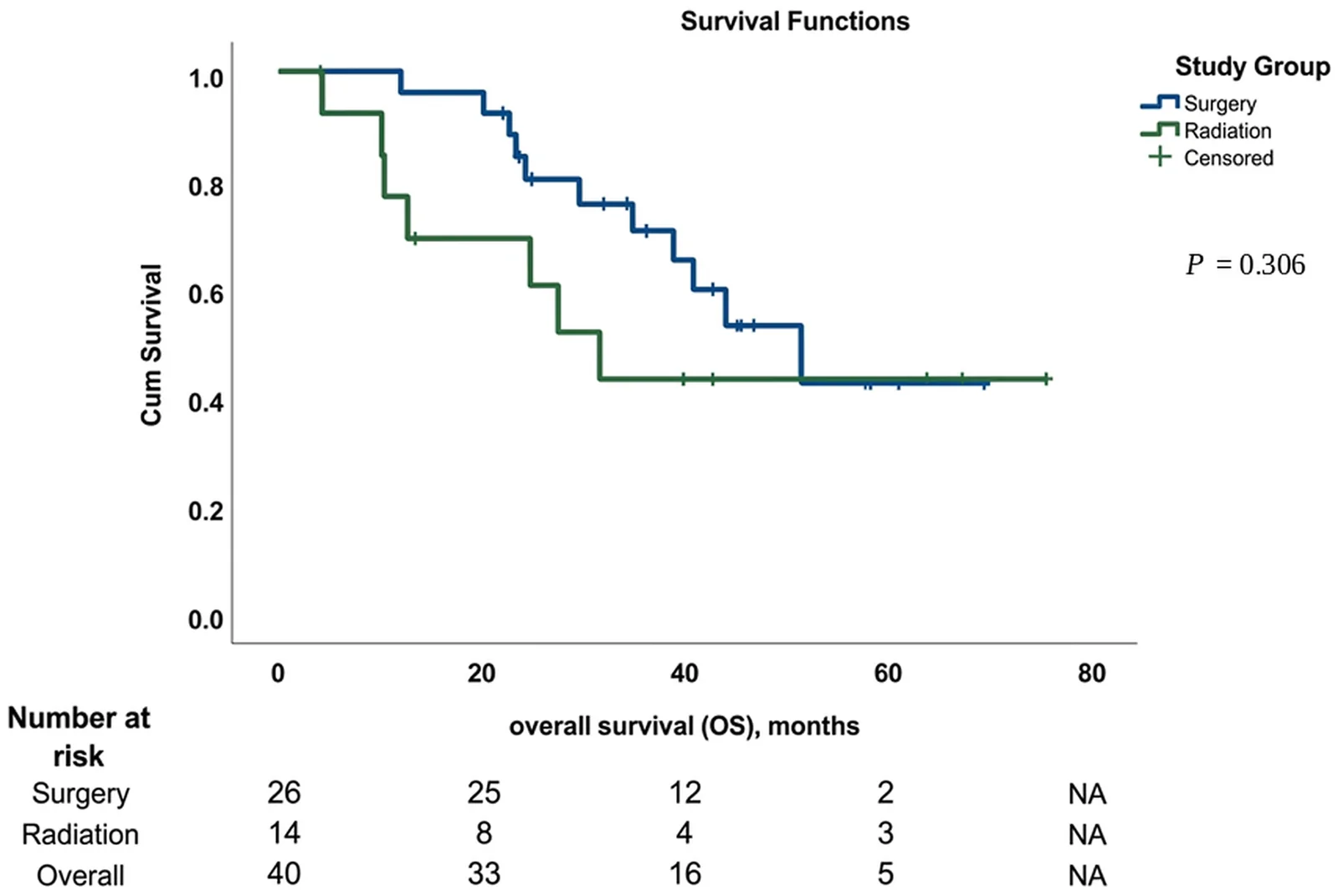
Overall survival according to treatment. Kaplan–Meier curve demonstrating no significant difference in OS between the two study groups. cum = cumulative. *p* = 0.306.

The median OS time observed over the follow-up period was 44 months (95% CI 28.61–59.46 months) across all study participants. Among those who underwent surgical resection, the median OS was 51 months (95% CI: 35.63–67.23 months), whereas patients treated with SBRT exhibited a median OS of 32 months (95% CI: 20.57–42.69 months). Comparative analysis utilizing log-rank tests revealed no significant association between treatment modality and OS across study groups (*p* = 0.31) (Figure 3).

### 3.4. Progression-Free Survival but Not Overall Survival Is Increased in Patients with a Single Lobe vs. Multiple Lobes Involved

**Figure 4:**
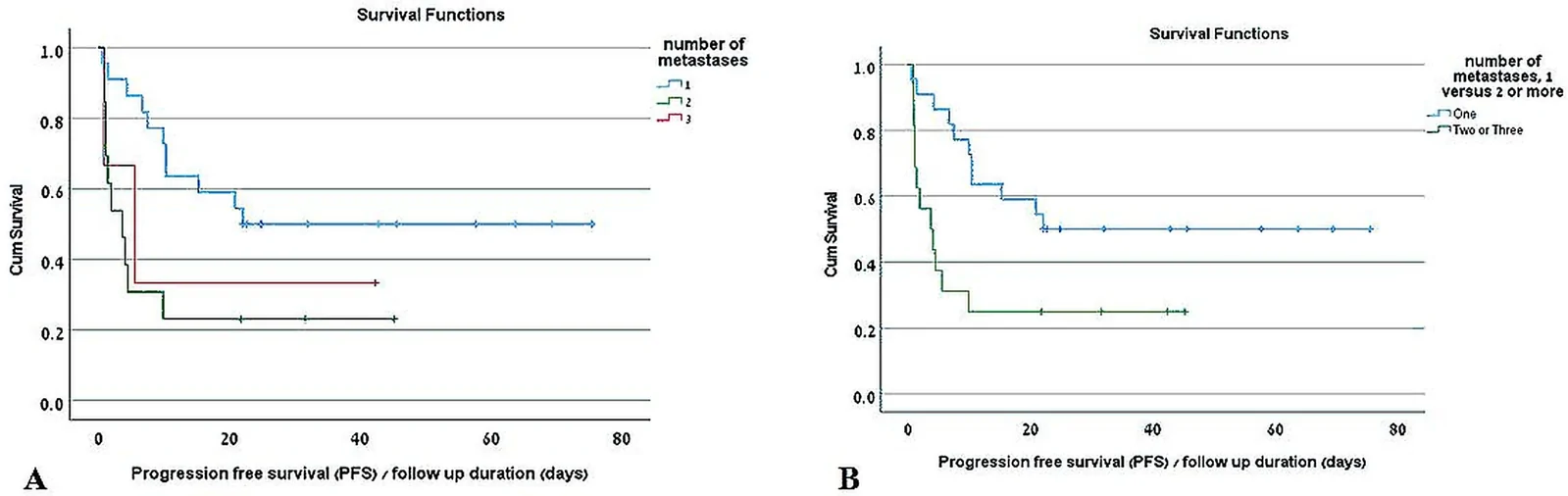
Progression-free survival by number of lobes involved. (**A**,**B**) Kaplan–Meier curves illustrating the impact of the number of lobes involved on progression-free survival. Patients were grouped by the number of lobes involved with tumor and then grouped into one, or two and more. A significantly worse PFS was observed among patients with more lobes involved. 1 = one lobe involved; 2 = two lobes involved; 3 = three lobes involved; cum = cumulative; one = one lobe involved; two or three = two or three lobes involved. (**A**) *p* = 0.041; (**B**) *p* = 0.013.

**Figure 5:**
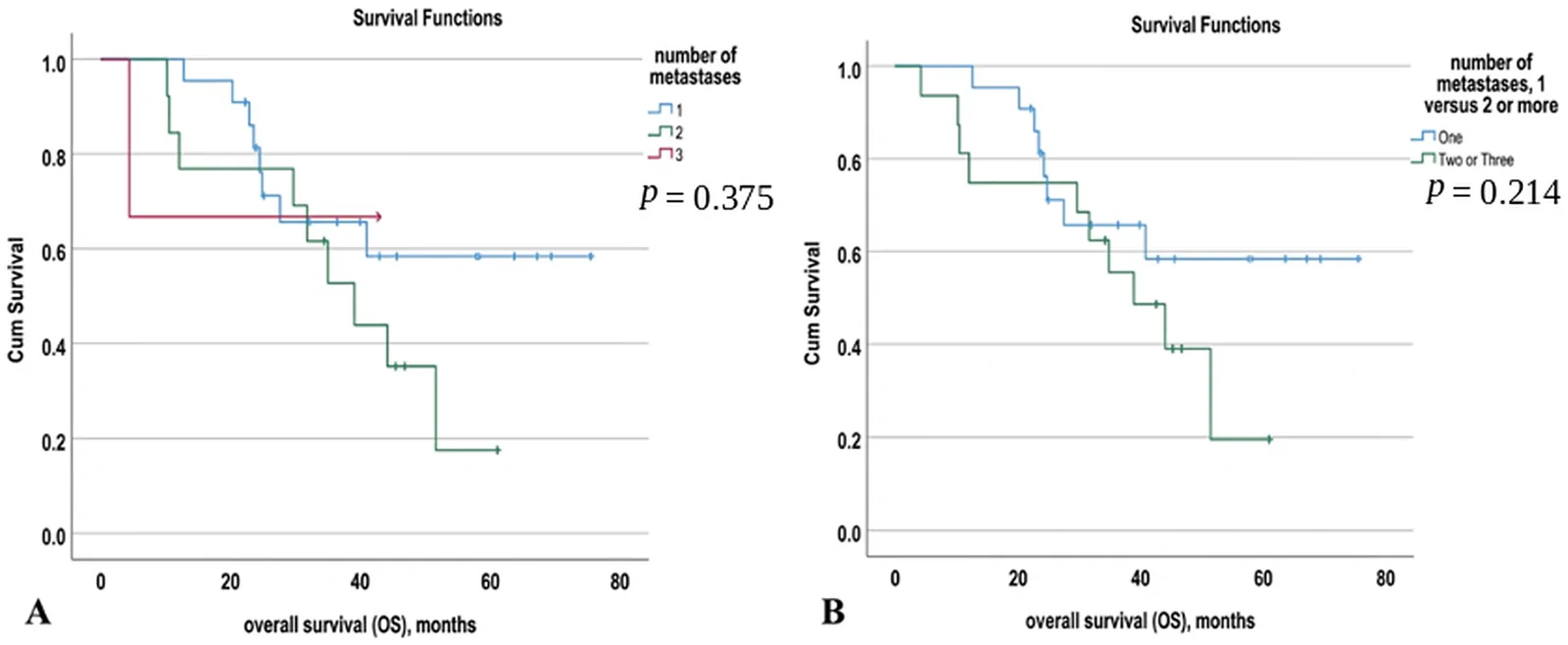
Overall survival by number of lobes involved. Kaplan–Meier curve demonstrating the effect of the number of involved lobes on overall survival. OS was not impacted by the number of lobes involved. 1 = one lobe involved; 2 = two lobes involved; 3 = three lobes involved; cum = cumulative; one = one lobe involved; two or three = two or three lobes involved. (**A**) *p* = 0.375; (**B**) *p* = 0.214.

To evaluate the impact of the number of lobes involved with metastases and their location on both PFS and OS, patients’ metastases were divided into three groups based on lobe involvement (1/2/3 lobes involved). The number of lobes involved is a significant factor affecting progression-free survival, as shown in Figure 4. Patients with more lobes involved experienced significantly worse PFS compared to those with involvement of only one lobe (*p* = 0.013). OS was not significantly affected, as shown in Figure 5. Taken together, local control increased in patients with a single lobe involved, without a significant impact on survival.

**Figure 6:**
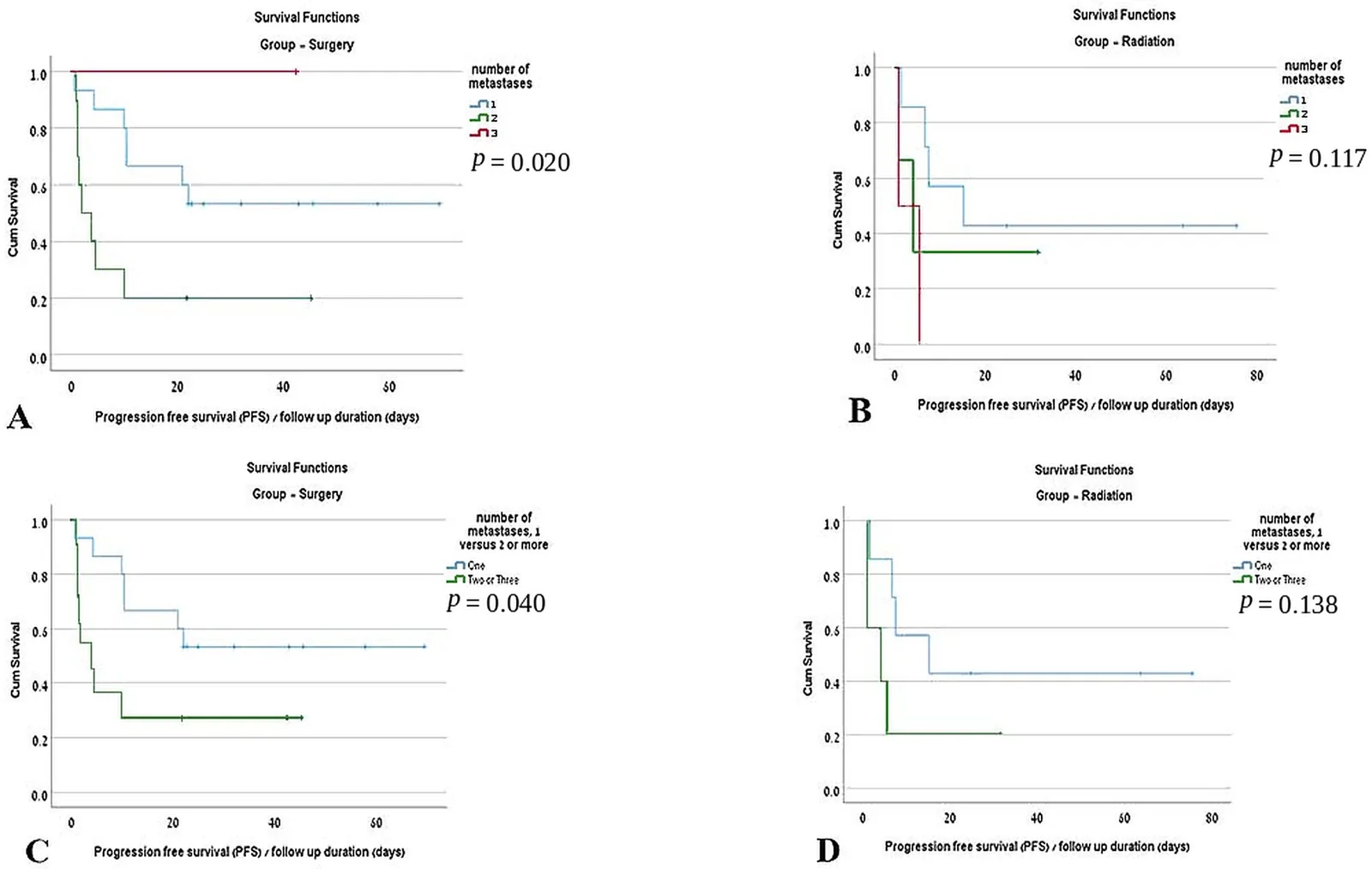
Progression-free survival by number of lobes involved in the different study groups. (**A**,**C**) Surgical resection group; (**B**,**D**) radiation group. Patients were grouped by the number of lobes involved with tumor and then grouped into one, or two and more. 1 = one lobe involved; 2 = two lobes involved; 3 = three lobes involved; cum = cumulative; one = one lobe involved; two or three = two or three lobes involved.

**Figure 7:**
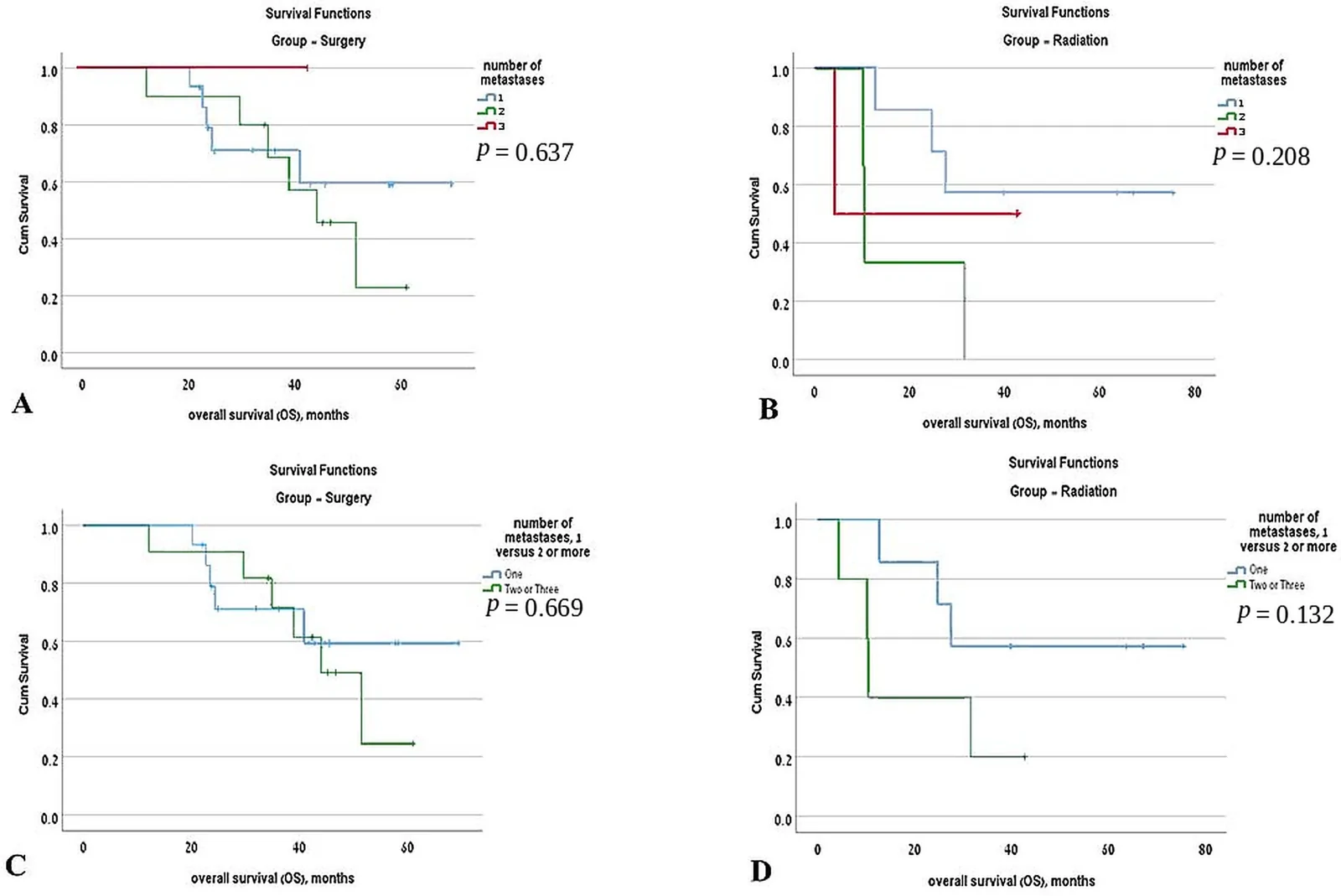
Overall survival by number of lobes involved in the different study groups. (**A**,**B**) Surgical resection group; (**C**,**D**) radiation group. 1 = one lobe involved; 2 = two lobes involved; 3 = three lobes involved; cum = cumulative; one = one lobe involved; two or three = two or three lobes involved.

We also investigated the impact of the number of lobes involved on progression-free survival and overall survival within the study groups as shown in Figure 6 and Figure 7. There was a significant difference in progression-free survival within the surgical study group, while no significant impact was observed in the radiation group.

We explored the relationship between the location of liver metastases and survival outcomes. We observed significantly less favorable PFS only in patients with metastases in the left lobe, while no other lobe showed any significant difference in PFS or OS, as shown in Table 3 and Table 4, respectively.

## 4. Discussion

Colorectal cancer remains a significant global health concern, ranking among the most commonly diagnosed cancers worldwide. CRLM management represents a substantial challenge in the clinical setting, impacting patients’ prognosis and treatment outcomes [1,2].

Our study aimed to assess the impact of treatment modality and liver metastasis site on PFS and OS in patients with CRLM. We analyzed 40 patients treated at Hadassah Medical Center between 2014 and 2024, comparing outcomes between surgical resection and SBRT. Additionally, we examined the influence of the number of involved lobes and specific anatomical locations on patient prognosis.

### 4.1. Treatment Modalities and Survival Outcomes

Consistent with previous literature, surgical resection remains the standard of care for patients with resectable liver metastases, offering the best potential for long-term survival [4,8,20,21]. In our cohort, patients who underwent surgical resection demonstrated a median OS of 51 months compared to 32 months in the SBRT group. However, despite a trend toward improved OS with surgery, the difference between the two treatment modalities did not reach statistical significance (*p* = 0.31), perhaps due to the small sample size of the cohort. Similarly, while median PFS was longer in the surgical group (10 months) compared to the SBRT group (8 months), this difference was not statistically significant (*p* = 0.468). As mentioned, these findings align with existing literature, which suggests that surgical resection is associated with improved survival outcomes.

SBRT represents a promising alternative for patients deemed unresectable or unfit for surgery due to comorbidities or lesion location, with a median OS of 16–46 months according to previous literature and our cohort [5]. The advent of stereotactic techniques has revolutionized radiation oncology, enabling precise delivery of ablative doses to individual liver lesions while minimizing radiation exposure to surrounding healthy tissue [16,22,23,24]. Importantly, in our cohort, SBRT appeared to yield similar results to surgical resection in terms of both PFS and OS, suggesting that SBRT may represent a viable alternative to surgery in patients not fit for surgery because of a more complex clinical situation. Further research is warranted in a prospective study. Notably, in our cohort the median BED was less than 100. This could affect the results we observed, based on a pooled analysis of outcomes with SBRT for liver metastases which suggests pushing for higher doses (BED > 130 Gy) to increase local control [3], especially since colon cancer cells are relatively more radioresistant than other solid tumors.

When choosing the treatment modality, careful patient selection and consideration of patients’ specific characteristics are important. In a retrospective analysis by Naoto et al., radiofrequency ablation and SBRT were compared with surgery among vulnerable patients, showing that SBRT could provide a safer option that spares patients with comorbidities from undergoing invasive procedures [25]. This could be a key advantage of SBRT over surgical resection.

Adverse effects are essential factors affecting treatment selection. Previous literature has reported differences in adverse effects. Surgical resection may be associated with surgical complications such as bleeding, infection, and prolonged hospitalization. In addition, post-operative morbidity and mortality are risks [26,27]. On the other hand, SBRT offers a non-invasive approach with fewer acute adverse effects and a shorter recovery period. Grade 2–3 hepatobiliary toxicity related to SBRT has been reported in up to 25% of patients, with severe adverse effects rarely observed [15,28].

Further research regarding the use of SBRT and systemic treatment specifically in colorectal liver metastases is warranted. Previous studies have shown that SBRT can enhance the efficacy of systemic treatment for metastatic disease more broadly. For instance, one study investigating the combination of focal radiation with PD-1 inhibitor immunotherapy in metastatic lesions found that SBRT reduced tumor cell resistance to treatment [29].

### 4.2. Impact of the Number of Involved Lobes on Prognosis

In this cohort there was a significant association between the number of lobes involved and PFS. Patients with involvement of two or more lobes experienced significantly worse PFS compared to those with metastases confined to a single lobe (*p* = 0.013). This suggests that local control is more challenging to achieve in patients with a greater hepatic tumor burden, likely due to the complexity of achieving complete tumor clearance while preserving adequate liver function. However, the number of lobes involved did not significantly impact OS (*p* = 0.214), indicating that while disease progression may occur more rapidly in these patients, their overall survival is influenced by other factors such as systemic therapy.

Additionally, in our study, the impact of the number of involved lobes on local control differed between treatment groups. Specifically, there was a significant difference in PFS between one and more than one lobes involved among patients who underwent surgical resection (*p* = 0.020), whereas no significant impact was observed in the SBRT group (*p* = 0.138). This finding suggests that surgical outcomes may be more affected by hepatic tumor burden, whereas SBRT outcomes might be influenced by other factors. Further research on a wider population is needed to understand this connection.

### 4.3. Anatomical Distribution of Metastases and Survival

Different factors affect the prognosis and treatment plans for CRLM, including the location of metastases inside the liver. Hallemeier et al. noted that the central liver near the hilum—where the biliary system, portal vein, and hepatic artery converge—is a more technically challenging metastasis location because of the complexity of the structures involved [3]. Although this concerns a different tumor with different characteristics, it is worth noting that a 2022 cohort study showed that in hepatocellular carcinoma, tumors in the anterolateral segments (segments 2, 3, 4b, 5, and 6) tend to have a more favorable prognosis than tumors in other segments, suggesting that the location of a tumor within the liver in CRC could also affect prognosis [6].

In our analysis, metastases located in the left lobe were associated with significantly worse PFS compared to other regions, suggesting that tumors in this location may have a higher tendency for progression. This observation is intriguing and warrants further investigation. It has been hypothesized that differences in vascular supply, lymphatic drainage, and adjacent organ involvement could contribute to differential outcomes based on metastatic location, as mentioned above [3]. However, no significant association was found between metastasis location and OS, reinforcing the notion that long-term survival is dictated by a combination of local and systemic disease control.

### 4.4. Limitations and Future Directions

Several limitations should be considered when interpreting our results. First, as a retrospective study, inherent selection biases may have influenced treatment decisions and outcomes. Second, our sample size was relatively small and confined to a single medical center, reducing the statistical power to detect significant differences in OS and PFS between treatment modalities. Future studies with larger, multi-institutional cohorts and prospective designs are needed to validate our findings and explore the biological mechanisms underlying the observed differences in PFS based on metastatic location.

## 5. Conclusions

Our findings emphasize the importance of individualized treatment planning for patients with CRLM. While surgical resection remains the gold standard, SBRT offers a viable alternative for patients deemed unsuitable for surgery. The observed correlation between greater hepatic tumor burden and poorer local control highlights the need for enhanced multimodal treatment approaches, such as combining SBRT with systemic therapy or other liver-directed interventions. Furthermore, the potential prognostic implications of metastatic location suggest that future treatment strategies may benefit from accounting for anatomical differences. Further research, including prospective trials, is warranted on CRLM management.

## Figures and Tables

**Table 1:** Baseline and pretreatment patient characteristics.

Variable	Study Population (N = 40)
Age (median, range, in years)	58.5 (29–90)
Sex	
Male (%)	28 (70)
Female (%)	12 (30)
Study group	
Surgery (%)	26 (65)
SBRT (%)	14 (35)
Location of metastases	
Caudate lobe (%)	3 (7.5)
Right lobe (%)	34 (85)
Left lobe (%)	22 (55)
Number of lobes involved	
One lobe (%)	24 (60)
Two or more lobes (%)	16 (40)
Follow-up time (median, range, in months)	46.24 (22.40–153.40)
Synchronous/metachronous metastases	
Synchronous (%)	9 (22.5)
Metachronous (%)	31 (77.5)
Surgery subgroup	
Age (median, range, in years)	59.5 (29–81)
Sex	
Male (%)	16 (61.5)
Female (%)	8 (38.5)
Location of metastases	
Caudate lobe (%)	1 (3.8)
Right lobe (%)	24 (92.3)
Left lobe (%)	13 (50)
Number of lobes involved	
One lobe (%)	15 (57.7)
Two or more lobes (%)	9 (42.3)
Synchronous/metachronous metastases	
Synchronous (%)	6 (23.08)
Metachronous (%)	20 (76.92)
SBRT subgroup	
Age (median, range, in years)	54 (33–90)
Sex	
Male (%)	10 (71.43)
Female (%)	4 (28.57)
Location of metastases	
Caudate lobe (%)	2 (14.29)
Right lobe (%)	10 (71.43)
Left lobe (%)	9 (64.29)
Number of lobes involved	
One lobe (%)	9 (64.29)
Two or more lobes (%)	5 (35.71)
Synchronous/metachronous metastases	
Synchronous (%)	3 (21.43)
Metachronous (%)	11 (78.57)

Table 1 describes the baseline characteristics for the overall study population (N = 40) and stratified by treatment subgroup (Surgery, n = 26; SBRT, n = 14). Values are reported as median (range) or count (%). SBRT = stereotactic body radiation therapy.

**Table 2:** Treatment features.

Variable	Study Population (N = 40)
Surgery	
Lobes resected	
Caudate lobe (%)	1 (3.8)
Right lobe (%)	21 (80.8)
Left lobe (%)	14 (53.8)
SBRT	
Cumulative radiation dose (median, range, Gy)	45 (33–55)
Dose of radiation per fraction (median, range, Gy)	9 (6.6–15)
Number of fractions	
3 (%)	1 (7.14)
5 (%)	13 (92.86)
BED (median, range, Gy)	85.5 (54.78–115.5)
Location of radiated metastases (n = 11)	
Caudate lobe (%)	1 (7.14)
Right lobe (%)	7 (50)
Left lobe (%)	5 (35.71)

Table 2 depicts surgical resection locations and SBRT dosing characteristics for the study cohort. Data is presented as median (range) or count (%). Gy = gray units; BED = biological effective dose.

**Table 3:** Progression-free survival by lobar distribution.

Event	N for Patients for Event (%)	N for Patients for No Event (%)	Median PFS for Event (Months)	Median PFS for No Event (Months)	*p*-Value
General					
Right lobe	34 (85)	6 (15)	9.95	10.41	0.918
Left lobe	22 (55)	18 (45)	5.55	NA	0.024
Caudate lobe	3 (7.5)	37 (92.5)	5.55	10.41	0.629
Surgery					
Right lobe	24 (92.31)	2 (7.69)	20.96	10.42	0.54
Left lobe	13 (50)	13 (50)	4.50	NA	0.03
Caudate lobe	1 (3.85)	25 (96.15)	NA	NA	0.33
SBRT					
Right lobe	9 (75)	3 (25)	7.89	10.42	0.70
Left lobe	4 (33.33)	8 (66.67)	5.85	15.64	0.33
Caudate lobe	2 (16.67)	10 (83.33)	NA	NA	0.051

Table 3 describes the effect of the lobar distribution of the metastases on progression-free survival. Each row shows a comparison between the median progression-free survival in patients with metastases localized to a specific lobe against those without metastases in the same lobe, using the log-rank test for statistical analysis. Instances where the test was inapplicable are denoted as “NA”, indicating cases where all patients in at least one group were censored before the end of the follow-up period. PFS = progression-free survival; NA = not applicable.

**Table 4:** Overall survival by lobar distribution.

Event	N for Patients for Event (%)	N for Patients for No Event (%)	Median OS for Event (Months)	Median OS for No Event (Months)	*p*-Value
General					
Right lobe	34 (85)	6 (15)	44.04	NA	0.997
Left lobe	22 (55)	18 (45)	38.88	NA	0.211
Caudate lobe	3 (7.5)	37 (92.5)	NA	44.04	0.835
Surgery					
Right lobe	24 (92.31)	2 (7.69)	NA	NA	0.67
Left lobe	13 (50)	13 (50)	NA	NA	0.90
Caudate lobe	1 (3.85)	25 (96.15)	NA	NA	0.57
SBRT					
Right lobe	9 (75)	3 (25)	NA	NA	0.65
Left lobe	4 (33.33)	8 (66.67)	74.71	105.66	0.59
Caudate lobe	2 (16.67)	10 (83.33)	NA	NA	0.28

Table 4 describes the effect of the lobar distribution of the metastases on overall survival. Each row compares the median OS in patients with metastases localized to a specific lobe against those without metastases in the same lobe, using the log-rank test for statistical analysis. OS = overall survival; NA = not applicable.

## Data Availability

The data presented in this study are available on request from the corresponding authors, due to restrictions related to patient privacy.

## Abbreviations

The following abbreviations are used in this manuscript:

CRLM	Colorectal cancer liver metastasis
SBRT	Stereotactic body radiotherapy
RT	Radiation therapy
PFS	Progression-free survival
OS	Overall survival
RILD	Radiation-Induced Liver Disease
BED	Biological effective dose
HR	Hazard ratio
CI	Confidence interval
IRB	Institutional Review Board
